# Heme oxygenase‐1 induction attenuates senescence in chronic obstructive pulmonary disease lung fibroblasts by protecting against mitochondria dysfunction

**DOI:** 10.1111/acel.12837

**Published:** 2018-10-19

**Authors:** Benjamin Even, Sarah Fayad‐Kobeissi, Jean‐Marie Gagliolo, Roberto Motterlini, Jorge Boczkowski, Roberta Foresti, Maylis Dagouassat

**Affiliations:** ^1^ Inserm U955, Equipe 04 Créteil France; ^2^ Université Paris Est Faculté de Médecine Créteil France; ^3^ Inserm U955, Equipe 12 Créteil France; ^4^ AP‐HP, Hôpital Henri Mondor, DHU A‐TVB, Antenne de Pneumologie Service de Réanimation Médicale Créteil France

**Keywords:** chronic obstructive pulmonary disease, fibroblasts, heme oxygenase‐1, mitochondria, mitophagy, senescence

## Abstract

Chronic obstructive pulmonary disease (COPD) is associated with lung fibroblast senescence, a process characterized by an irreversible proliferation arrest associated with secretion of inflammatory mediators. ROS production, known to induce senescence, is increased in COPD fibroblasts and mitochondria dysfunction participates in this process. Among the battery of cellular responses against oxidative stress damage, heme oxygenase (HO)‐1 plays a critical role in defending the lung against oxidative stress and inflammation. Therefore, we investigated whether pharmacological induction of HO‐1 by chronic hemin treatment attenuates senescence and improves dysfunctional mitochondria in COPD fibroblasts. Fibroblasts from smoker controls (S‐C) and COPD patients were isolated from lung biopsies. Fibroblasts were long‐term cultured in the presence or absence of hemin, and/or ZnPP or QC‐15 (HO‐1 inhibitors). Lung fibroblasts from smokers and COPD patients displayed in long‐term culture a senescent phenotype, characterized by a reduced replicative capacity, an increased senescence and inflammatory profile. These parameters were significantly higher in senescent COPD fibroblasts which also exhibited decreased mitochondrial activity (respiration, glycolysis, and ATP levels) which led to an increased production of ROS, and mitochondria biogenesis and impaired mitophagy process. Exposure to hemin increased the gene and protein expression level of HO‐1 in fibroblasts and diminished ROS levels, senescence, the inflammatory profile and simultaneously rescued mitochondria dysfunction by restoring mitophagy in COPD cells. The effects of hemin were abolished by a cotreatment with ZnPP or QC‐15. We conclude that HO‐1 attenuates senescence in COPD fibroblasts by protecting, at least in part, against mitochondria dysfunction and restoring mitophagy.

## 1 INTRODUCTION

Chronic obstructive pulmonary disease (COPD) represents the fourth leading cause of death worldwide (Mannino & Buist, 2007). This pathologic state is characterized by airways remodeling and destruction of the alveolar wall, a condition called emphysema. Both processes lead to an irreversible airflow obstruction, which is the hallmark of the disease. Cigarette smoke (CS) exposure is the main risk factor for COPD (Yoshida & Tuder, 2007). CS contains a high concentration of reactive oxygen species (ROS, 10^17^/puff) and thus plays a major role in promoting oxidative stress in COPD (Church & Pryor, 1985), (Carnevali et al., 2003). Oxidative stress provokes inflammation and participates in the emergence of cell senescence, another major feature of this disease (Barnes, 2017).

Cell senescence can stem from shortening of telomeres during continuous cell replication (replicative senescence) or be triggered by other stressors, such as oxidative stress inducers (hydrogen peroxide, CS) or inflammatory mediators (premature senescence; Nyunoya et al., 2009). These stimuli can act through two main different pathways: p53‐p21 and p16‐retinoblastoma protein (pRb), respectively (Campisi, 2005). In the lung, cell senescence leads to a decreased regenerative capacity and an increased cytokine production by structural cells (epithelium, fibroblasts) through a senescence‐associated secretome (Amsellem et al., 2011; Dagouassat et al., 2013; Noureddine et al., 2011; Tsuji, Aoshiba, & Nagai, 2010). However, other mechanisms, including mitochondrial dysfunction and defective mitophagy, may also contribute to ROS‐mediated senescence in COPD (Lerner, Sundar, & Rahman, 2016).

Mitophagy is a protective mechanism which minimizes the proportion of dysfunctional mitochondria. This mechanism involves the stabilization of PTEN‐induced putative kinase 1 (PINK‐1) on the outer membrane of mitochondria following the loss of ΔΨm (Matsuda et al., 2010). The E3 ligase Parkin is then recruited to catalyze K63‐linked ubiquitination of outer membrane proteins. This event facilitates the targeting of mitochondria to form autophagosomes (Hattori, Saiki, & Imai, 2014). PINK1 and Parkin knockout mice exhibit increased ROS levels and dysfunctional mitochondria (Gispert et al., 2009; Palacino et al., 2004) and bronchial epithelial deleted from Parkin or PINK1 exposed to CS display an enhanced mtROS production and cellular senescence (Ito et al., 2015). These results reinforce the role of oxidative stress as an inducer of cell senescence upon CS exposure and suggest that an impaired mitophagy could be a mechanism inducing ROS‐mediated cell senescence in COPD.

Indeed, defective mitophagy has been already involved in lung cellular senescence upon exposure to CS in vitro and in vivo (Ahmad et al., 2015).

Among the battery of cellular responses against oxidative stress damage, heme oxygenase (HO)‐1, the inducible isoform of heme oxygenase, plays a critical role in defending the lung against inflammatory and oxidant‐induced cellular and tissue injury. HO‐1 catalyzes heme degradation to produce equimolar amounts of carbon monoxide (CO), which exhibits anti‐inflammatory properties, and biliverdin which is converted to bilirubin, a powerful antioxidant, by biliverdin reductase. In addition, HO‐1 and CO induce mitochondrial biogenesis (Piantadosi et al., 2011) and recent findings indicate that this system is also implicated in mitochondrial quality control (Hull et al., 2016). The expression of HO‐1 is induced in smokers with mild COPD as compared to smoker controls (Maestrelli et al., 2001), revealing a potential protective role of this enzyme against oxidative stress‐mediated cell senescence and mitochondrial dysfunction in COPD. However, to the best of our knowledge, this hypothesis has not been examined yet.

Therefore, in the present study, we investigated whether pharmacological induction of HO‐1 by chronic hemin treatment attenuates senescence and rescues an impaired mitophagy of pulmonary fibroblasts from COPD patients.

## 2 RESULTS

### 2.1 Patient’s clinical and demographic features

Results from fibroblasts from COPD patients were compared with those obtained in fibroblasts from smokers without COPD (S‐C group). The clinical and demographic features of the subjects in the two groups are presented in Table 1. The two groups were similar in age and smoking history. Subjects with COPD displayed a mild to moderate degree of disease as revealed by GOLD stages I and II (4 and 10 subjects, respectively) and an emphysema score of 45.6 ± 4.5 over 100 (Bankier, De Maertelaer, Keyzer, & Gevenois, 1999).

**Table 1:** Patient characteristics

	Smokers (S‐C)	COPD smokers	*p *value
Patients (*n*)	13	14	
Age (years)	57 (48–67)	60 (51–74)	ns
Sex (M/F)	6/7	7/7	
FEV_1_/VC	81 (72/107)	61 (42/69)***	<0.001
FEV_1_ (%)	89 (76–113)	73 (50–113)**	<0.001
GOLD (*n*) 0/I/II/III/IV	13/0/0/0/0	0/4/10/0/0	
Smoking history (pack‐yrs)	43 (30–60)	46 (20–75)	ns
Emphysema score (%)	12.9 ± 3.5	45.3 ± 4.6**	<0.001

Notes

COPD: chronic obstructive pulmonary disease; GOLD: Global Initiative for Chronic Obstructive Lung Disease; VC: vital capacity; FEV_1_, forced expiratory volume in 1 s.

Emphysema score was assessed on 0%–100% scale.

Data are expressed as median with minimum and maximum quartiles (in parentheses), except for emphysema score, which is expressed as mean ± SEM.

**p < 0.01.

***p < 0.001 COPD patients vs. smokers.

### 2.2 Hemin induces gene, protein expression, and activity of HO‐1

**Figure 1:**
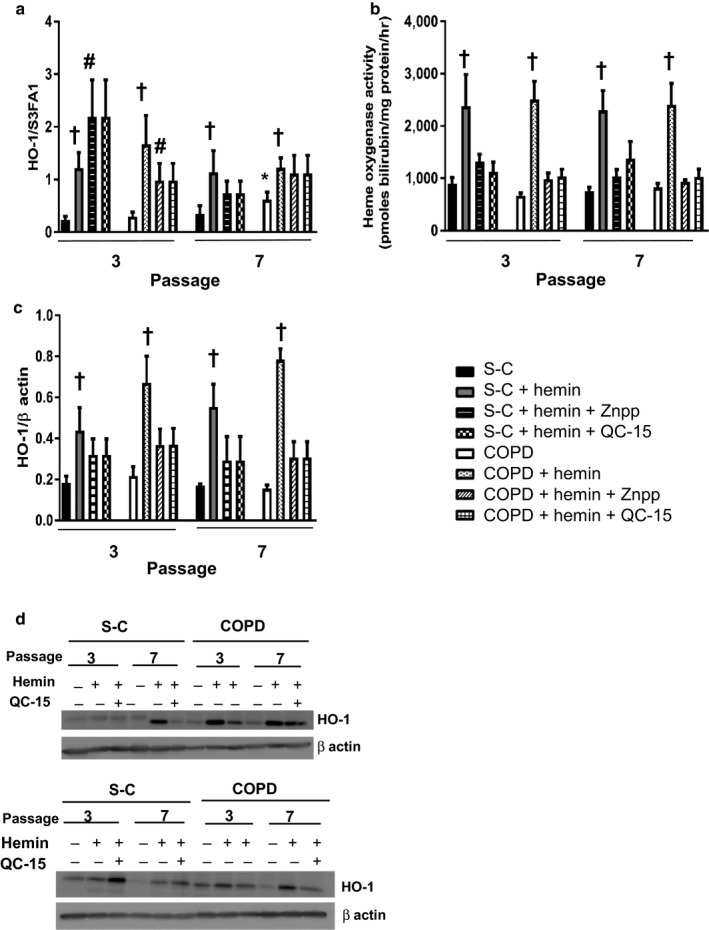
Hemin induces gene, protein expression, and activity of heme oxygenase 1 (HO‐1). Fibroblasts from COPD patients (*n* = 14) and smoker controls (S‐C, *n* = 13) were treated chronically with either hemin (10 µM) alone or in the presence of inhibitors of HO‐1 activity: ZnPPIX (1 µM) or QC‐15 (15 µM) for 4 weeks. (a) Quantitative transcriptional expression of *HMOX1* by real‐time qPCR. (b) Heme oxygenase activity. (c) Quantification of HO‐1 protein expression by western blot. (d) Two examples of western blot. Data are presented as mean ± *SEM* in the whole Figure. *p < 0.05 passage 7 (senescent stage) vs. passage 3 (nonsenescent stage), ^†^ *p* < 0.05, ^††^ *p* < 0.01 cells treated with hemin vs. cells treated with DMSO, ^#^ *p* < 0.05 cells treated with inhibitors of HO‐1 activity vs. cells treated with solvent

To investigate the role of hemin and HO‐1 in counteracting fibroblasts senescence, we established a protocol for long‐term exposure of cells to nontoxic concentrations of hemin. We found that cells treated every two days with hemin exhibited higher *HMOX1* gene expression (Figure 1a), which was reflected in an increased level of heme oxygenase activity (Figure 1b) and HO‐1 protein expression (Figure 1c,d). The increase in HO‐1 was evident in nonsenescent (P3) and senescent cells (P7) and was not significantly different between cells derived from S‐C or COPD subjects. In parallel experiments, cells were treated with hemin in the presence of heme oxygenase inhibitors ZnPPIX or QC‐15 and, while the two compounds did not change the levels of *HMOX1* gene expression compared with hemin alone (Figure 1a), they completely abolished the hemin‐mediated increase in heme oxygenase activity (Figure 1b). We note that at P3 and in the absence of hemin treatment, the *HMOX1* expression level was not different between fibroblasts from COPD patients and from S‐C. At P7, *HMOX1* expression was higher in fibroblasts from COPD patients as compared to S‐C but no difference in heme oxygenase activity and HO‐1 protein expression was observed (Figure 1a,b,c).

### 2.3 Hemin prevents replicative senescence in fibroblasts from copd patients

**Figure 2:**
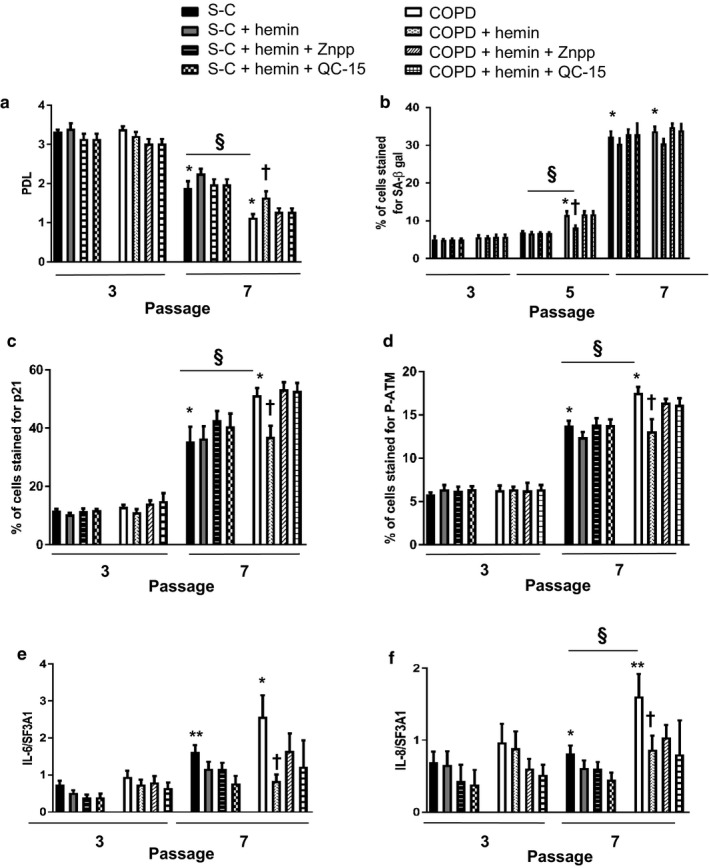
Hemin attenuates replicative senescence in COPD fibroblasts. Fibroblasts from COPD patients (*n* = 14) and smoker controls (S‐C, *n* = 13) were treated chronically with either hemin (10 µM) alone or in the presence of inhibitors of HO‐1 activity: ZnPPIX (1 µM) or QC‐15 (15 µM) for 4 weeks. (a) The rate of proliferation was evaluated by the population doubling level (PDL). (b) Percentage of senescence‐associated (SA) β‐gal‐positive cells. (c, d) Quantification of p21 and P‐ATM obtained by immunostaining. Transcriptional expression of inflammatory mediators (IL‐6, IL‐8). (e, f) by real‐time qPCR in pulmonary fibroblasts. Data are presented as mean ± SEM in the whole Figure. *p < 0.05, **p < 0.01 passage 7 (senescent stage) vs. passage 3 (nonsenescent stage), ^†^ *p* < 0.05 cells treated with hemin vs. cells treated with DMSO, ^§^ *p* < 0.05 COPD vs. S‐C

We next examined whether chronic exposure to hemin could attenuate replicative senescence and the secretome associated with the senescence phenotype. Fibroblasts from COPD patients were not different from S‐C at nonsenescent passage, but displayed senescence hallmarks at senescence passage: a lower senescence‐associated PDL (Figure 2a), an increased SA β‐gal staining (Figure 2b), and increased p16 and IL‐8 mRNA expression (Supporting Information Figures S1, and S2f) and increased p16 protein expression (S1), as compared to S‐C. In contrast, COPD fibroblasts showed an increase in p21 and P‐ATM protein expression which was higher than in smoker fibroblasts (Figure 2c). In addition, IL‐6 mRNA expression was similar to that of S‐C (Figure 2f). It has to be noted that the difference between COPD and S‐C in terms of SA β‐gal staining was evident at P5 (Figure 2b), whereas that concerning p16 and PDL was visible at P7 (Figure 2a, and Supporting Information Figure S1). Such dissociation between markers of senescence was reported previously and could be related to diverse mechanisms underlining the evolution of senescence. Repeated exposures of fibroblasts to hemin protected against replicative senescence. Indeed, hemin further increased senescence‐associated PDL at P7 (Figure 2a) and decreased the induction of SA β‐gal (Figure 2b), p21 (Figure 2c), p16 (Supporting Information Figure S1), P‐ATM (Figure 2d), IL‐6, and IL‐8 (Figure 2e,f). These effects were more pronounced in fibroblasts from COPD patients than from the S‐C group and were abolished in cells exposed to hemin in the presence of ZnPPIX or QC‐15 (Figure 2).

### 2.4 Hemin reduces ROS production linked to mitochondrial dysfunction

**Figure 3:**
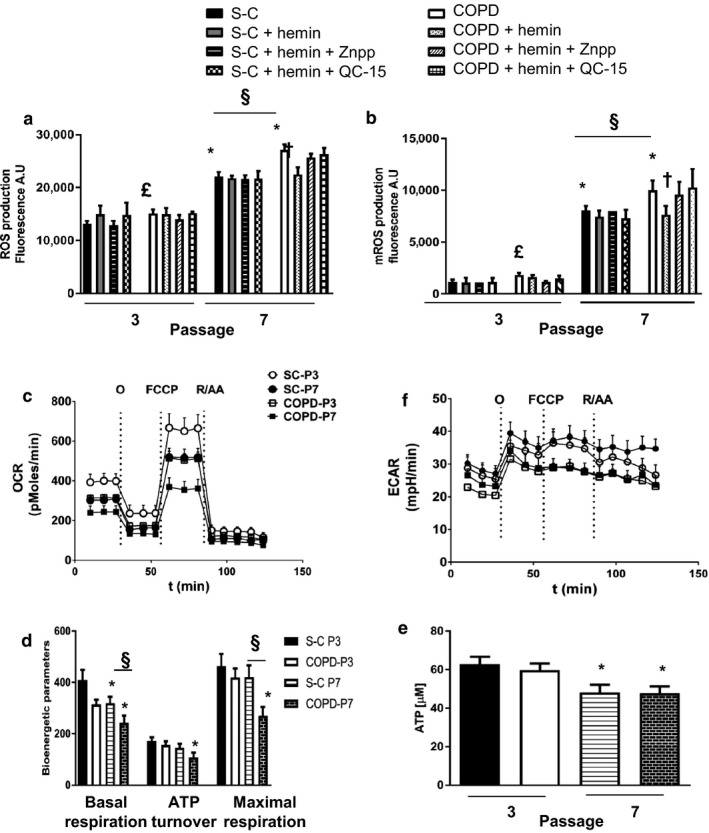
Hemin decreases total ROS and mROS linked to mitochondrial dysfunction Fibroblasts from COPD patients (*n* = 14) and smoker controls (S‐C, *n* = 13) were treated chronically with either Hemin (10 µM) alone or with inhibitors of HO‐1 activity: ZnPPIX (1 µM) or QC‐15 (15 µM) for 4 weeks. (a) Production of ROS was measured by DCFH‐DA fluorescence. Values are expressed as the percentage of control (ratio of fluorescence at 90 min over T0). (b) Production of mitochondrial ROS was measured by MitoSOX fluorescence. Values are expressed as the percentage of control (ratio of fluorescence at 15 min over T0). (c, d,) Bioenergetic parameters and extracellular acidification rate (ECAR, an index of glycolysis (f)) were measured using the Seahorse XF analyzer. (e) ATP levels, *n* = 7 in each group. Data are presented as mean ± SEM in the whole Figure. *p < 0.05 passage 7 (senescent stage) vs. passage 3 (nonsenescent stage), ^§^ *p* < 0.05 COPD passage 7 (vs. S‐C at passage 7, £*p* < 0.05 COPD at passage 3 vs. S‐C at passage 3, ^†^ *p* < 0.05 cells treated with hemin vs. cells treated with DMSO, ^§^ *p* < 0.05 COPD vs. S‐C

Since ROS play a major role in the induction of senescence and are increased in COPD (Barnes, 2017), we next investigated whether chronic exposure to hemin attenuated ROS production and analyzed cellular or mitochondrial ROS levels in fibroblasts from COPD and S‐C patients. Intracellular ROS production was statistically higher in fibroblasts from COPD patients as compared to controls at nonsenescent passage (Figure 3a), and replicative senescence (P7) was associated with a further increase in ROS levels in both COPD and S‐C fibroblasts. However, even in the senescent passage, fibroblasts from COPD displayed higher ROS levels than cells from S‐C (Figure 3a). We also observed a strongly enhanced mitochondrial ROS production at senescent passage, and again, we confirmed the differences between COPD and S‐C fibroblasts already noted for cellular ROS levels (Figure 3b). In order to confirm whether mitochondrial ROS play a role in the induction of fibroblast senescence, we treated cells with mitoquinol (an antioxidant which accumulates in mitochondria). Repeated exposures of COPD fibroblasts to mitoquinol reversed at senescent stage the lower PDL associated with senescence, and decreased the induction of SA β‐gal and p21 compared with untreated COPD fibroblasts (Supporting Information Figure S6). Moreover, the exposure to mitoquinol abolished the difference between fibroblasts from COPD patients and S‐C group. No significant effects were observed in S‐C fibroblasts after treatment with mitoquinol. These data indicated that ROS, and especially mitochondrial ROS, are a factor associated with the senescent phenotype in S‐C and COPD fibroblasts.

Since higher mitochondrial ROS suggests mitochondrial dysfunction, we investigated cellular metabolism and mitochondrial function by measuring respiration, glycolysis, and ATP levels in S‐C and COPD cells at P3 and P7. At nonsenescent passage, we observed a general depression in oxygen consumption in fibroblasts from COPD patients compared to S‐C, with a decrease in basal and maximal respiration (Figure 3c,d and Supporting Information Figure S2). At senescent stages, these differences were exacerbated (Figure 3c,d) but we highlight the fact that also fibroblasts from S‐C at P7 exhibited an inhibited mitochondrial function compared to S‐C cells at P3. A similar profile was detected for glycolysis (ECAR, Figure 3f), emphasizing that the metabolic status of COPD fibroblast was compromised even at nonsenescent passage. Accordingly, ATP levels were lower in COPD fibroblasts at P3 and were considerably decreased in both S‐C and COPD cells at P7 (Figure 3e), as expected for senescence stages. These data suggest that senescence alone, whether occurring in S‐C or COPD fibroblasts, is accompanied by decreased mitochondrial activity, that this phenomenon is exacerbated in COPD as compared to S‐C fibroblasts and that the increased production of ROS in fibroblasts from COPD patients is linked to a mitochondrial dysfunction.

**Figure 4:**
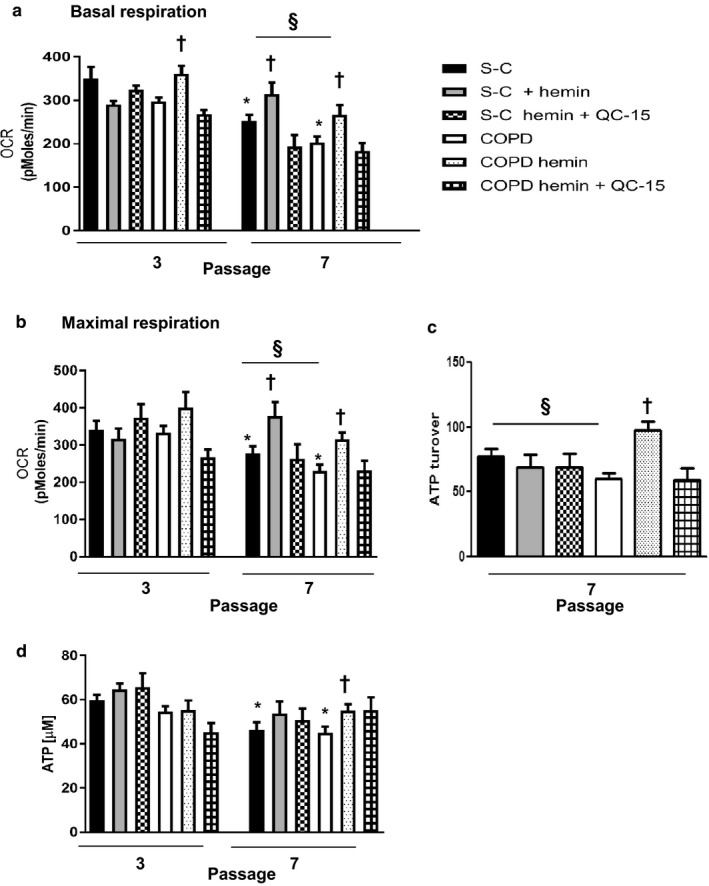
Hemin protects against mitochondria dysfunction. Fibroblasts from COPD patients (*n* = 14) and smoker controls (S‐C, *n* = 13) were treated chronically with either Hemin (10 µM) alone or with an inhibitor of HO‐1 activity: QC‐15 (15 µM) for 4 weeks. Bioenergetic parameters (a) basal respiration, (b) maximum respiration, (c) ATP turnover were measured using the Seahorse XF analyzer. (d) ATP levels. Data are presented as mean ± SEM in the whole Figure. *p < 0.05 passage 7 (senescent stage) vs. passage 3 (nonsenescent stage), ^†^ *p* < 0.05 cells treated with hemin vs. cells treated with DMSO, ^§^ *p* < 0.05 COPD vs. S‐C

As observed for replicative senescence, repeated exposure to hemin decreased cellular and mitochondrial ROS production in COPD fibroblasts (Figure 3a,b), with a parallel recovery in mitochondrial respiration, spare respiratory capacity, glycolysis, and ATP production at P7 (Figure 4a,b,c,d, and Supporting Information Figure S3). A similar improvement in respiration was also induced by hemin in S‐C senescent fibroblasts (Figure 4a,b). These effects were abolished by concomitant administration of hemin with either ZnPPIX or QC‐15 (Figures 3a,b, and 4). Interestingly, while cotreatment of hemin with QC‐15 prevented the rescue of mitochondrial respiration in COPD senescent fibroblasts, QC‐15 did not affect bioenergetic parameters and glycolysis (S4, data not shown), implying that the effect of hemin and HO‐1 induction is more specific toward modulation of mitochondrial function.

### 2.5 Hemin modulates mitochondrial biogenesis and improves mitophagy in COPD fibroblasts

**Figure 5:**
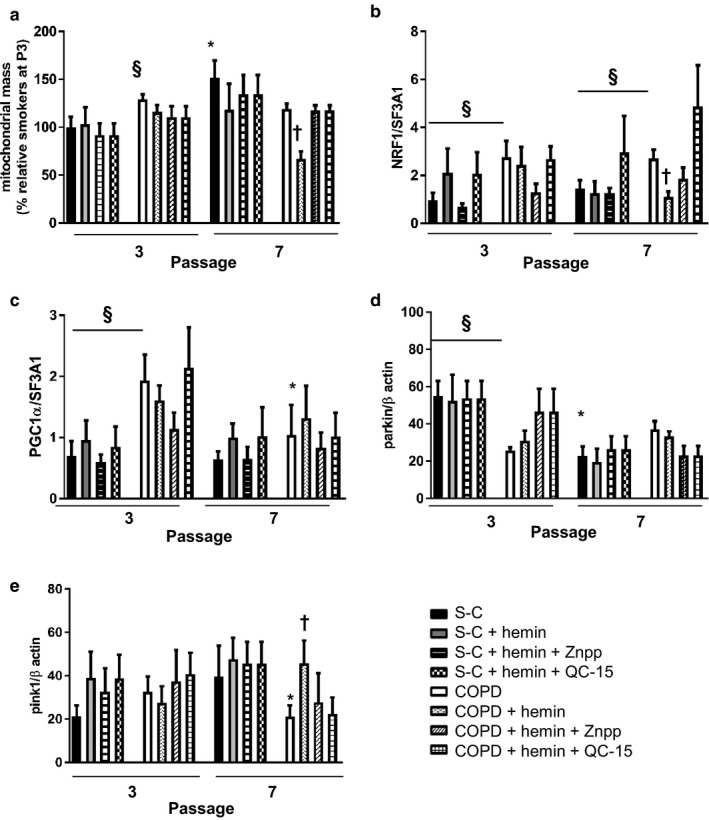
Hemin decreases the biogenesis of mitochondria and increases mitophagy in COPD fibroblasts. Pulmonary fibroblasts derived from COPD patients (*n* = 14) and smoker controls (S‐C, *n* = 13) were treated chronically with either hemin (10 µM) alone or in the presence of inhibitors of heme oxygenase activity: ZnPPIX (1 µM) or QC‐15 (15 µM). (a) Mitochondria mass detection was performed by using acridine orange 10‐nonyl bromide staining. Cells were analyzed with flow cytometry. (b, c) Quantitative transcriptional expression of gene involved in biogenesis (NRF‐1 and PGC1α) by real‐time qPCR in pulmonary fibroblasts. (d, e) Expression of proteins involved in mitophagy (PARKIN and PINK‐1) by western blot. Data are presented as mean ± SEM in the whole Figure. *p < 0.05, passage 7 (senescent stage vs. passage 3 (nonsenescent stage), ^†^ *p* < 0.05 cells treated with hemin vs. cells treated with DMSO, ^§^ *p* < 0.05 COPD vs. S‐C

To further investigate the mechanisms that contribute to the improvement of mitochondria function by hemin, we analyzed mitophagy and mitochondrial biogenesis, two related cell pathways that may be affected by senescence and COPD. We first assessed mitochondrial content in S‐C and COPD fibroblasts at nonsenescent and senescent stages. We found that mitochondrial mass was higher in fibroblasts from COPD patients as compared to S‐C at nonsenescent passage (Figure 5a). Mitochondrial mass significantly increased in S‐C fibroblasts at senescent passage, whereas it remained unchanged in COPD fibroblasts. As a consequence, the difference observed between the two groups before senescence was lost at the senescence passage (Figure 5a). In addition, we evaluated autophagy by determining the expression of p62, LC3I, and II (Supporting Information Figure S5). The ratio LC3II/I was not modified whatever the fibroblasts or treatment considered. Concerning the expression of p62, we noted a slight but not significant increase in COPD fibroblasts at nonsenescent stage. However, this effect was lost at senescent passage. Hemin did not modify the expression level of these three proteins. These different experiments show that the autophagic flow is not apparently modified in fibroblasts from smokers and COPD patients. However, staining with an antibody against LC3 (an autophagosome marker) colocalized with MitoSOX Red (used as a marker of mitochondria) in fibroblasts from COPD patients at senescent stage indicated autophagosome accumulation (Supporting Information Figure S5c), confirming a defect of mitophagy in COPD fibroblasts. Hemin abolished this colocalization, suggesting a restoration of mitophagy in these cells. Interestingly, the mRNA expression of NRF1 and PGC‐1α (the master gene of mitochondrial biogenesis) was significantly higher in COPD fibroblasts compared with controls at nonsenescent passage, although this difference was less pronounced at senescent passage for PGC‐1α (Figure 5c,b). These results advocate for a compensatory effect of mitochondria biogenesis in fibroblasts from COPD patients at nonsenescent stage. To assess this idea, we treated fibroblasts with an inhibitor of mitochondrial biogenesis in long‐term culture. This treatment led to the death of cells from COPD patient, reinforcing this hypothesis (data not shown).

We analyzed mitophagy by examining the protein expression of Parkin and PINK‐1. We observed that protein expression of Parkin was dramatically decreased in COPD compared to S‐C fibroblasts at nonsenescent passage (Figure 5d). Parkin expression significantly decreased in S‐C fibroblasts at senescent passage, whereas it remained unchanged in COPD fibroblasts. No changes in PINK‐1 expression were observed in both groups at nonsenescent and senescent passages (Figure 5e).

Chronic exposure of cells to hemin led to a decrease in mitochondrial mass in COPD fibroblasts at senescent passage (Figure 5a). This effect was accompanied by lower NRF1 expression (Figure 5b) along with a significant increase in PINK1 protein expression (Figure 5e) and was not observed at nonsenescence stage or in S‐C cells. Importantly, cotreatment with ZnPPIX or QC‐15 completely abolished this response.

**Figure 6:**
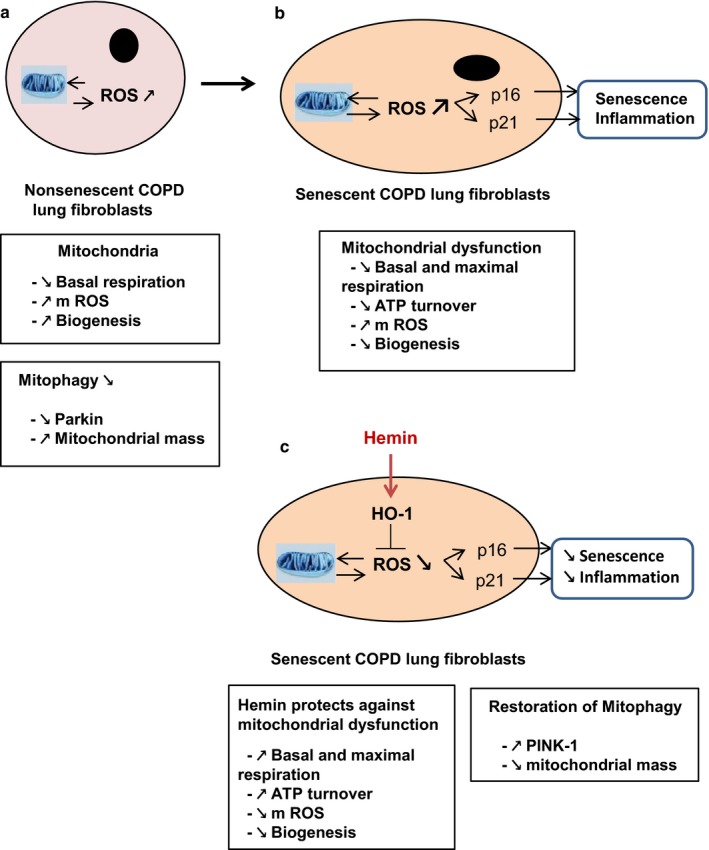
Senescence prevention by HO‐1 activation. (a) Mitochondrial dysfunction, characterized by a decrease in basal respiration and an increase in mROS production, is observed at nonsenescent passage in COPD lung fibroblasts. At the same time, defective mitophagy is also present (increase in mitochondria mass and decrease in Parkin), which ensures the maintenance of malfunctional mitochondria. To compensate for this effect, fibroblasts increase mitochondria biogenesis. (b) At the onset of senescence state, mitochondria dysfunction is exacerbated as evidenced by a further decrease in basal and maximal respiration, ATP turnover, and increased mROS production. However, mitochondria biogenesis compensatory pathway is lost. All of this contributes to the induction of senescence in senescent lung fibroblasts. (c) The induction of HO‐1 by hemin exerts a protective effect on mitochondria function (improvement of all parameters) and increases mitophagy (decrease in mitochondria mass and increase in PINK‐1). Thus, HO‐1 prevents the induction of senescence in COPD fibroblasts by protecting against mitochondria dysfunction

Collectively, these results suggest that COPD fibroblasts are characterized by an impaired mitophagy at nonsenescent passage as evidenced by an increased mitochondrial mass and compensatory mitochondrial biogenesis. This phenomenon persisted at senescent passage and was associated with increased senescence in COPD cells. Continuous exposure to nontoxic levels of hemin prevented these events by ameliorating mitochondrial function (Figure 6).

## 3 DISCUSSION

The main results of this study are the following: (a) At nonsenescent passage, compared to fibroblasts from control smokers, COPD fibroblasts display hallmarks of mitochondrial dysfunction (increased mitochondrial ROS production and decreased respiration, glycolysis, and ATP levels) accompanied by impaired mitophagy as evidenced by an increased mitochondrial mass, compensatory mitochondrial biogenesis, and decreased expression of Parkin protein; (b) these phenomena were aggravated at senescent stage in both smoker and COPD fibroblasts and were associated in this last group to an increased replicative senescence; and (c) hemin treatment attenuated mitochondrial dysfunction, restored mitophagy, and suppressed the increased senescence in COPD fibroblasts; these effects were abrogated by two different and not chemically related HO inhibitors. Collectively, these results demonstrate that senescence of pulmonary COPD fibroblasts is associated with mitochondrial dysfunction, suggesting a close relationship between an impaired mitophagy, accumulation of dysfunctional mitochondria producing excessive amount of ROS, and aggravation of replicative senescence. The protective effects of hemin against COPD fibroblasts senescence are likely the consequence of a primary effect of HO‐1 induction restoring the defective mitophagy of these cells.

Our results showing mitochondrial dysfunction in fibroblasts from COPD patients even at nonsenescent passage are consistent with data published in the literature showing that bronchial epithelial cells from COPD patients exhibited swollen, elongated mitochondria with fragmentation, and disruption of cristae (Hoffmann et al., 2013). Since fibroblasts were isolated from lung samples of patients suffering from lung cancer, a cancer‐related fibroblast phenotype could explain the differences between COPD and control smokers. However, we think this possibility unlikely because: (a) Smoker controls and COPD patients suffered from similar cancer histological types, (b) biopsies were harvested at a distance from the tumor and were verified to be free of malignant cells, and (c) primary cultures of lung fibroblasts did not exhibit Cancer Associated Fibroblasts markers (data not shown). Cigarette smoke can trigger mitochondrial dysfunction in COPD fibroblasts since lung epithelial cells and fibroblasts exposed to cigarette smoke exhibit an increase in mitochondrial ROS, reduced ATP levels, and changes in mitochondria structure (Ahmad et al., 2015), (Hoffmann et al., 2013). However, other factors probably contribute to mitochondrial dysfunction in COPD cells since in the present study, the smoking history of COPD and control smokers was similar. A COPD specific impaired mitophagy could be one of these mechanisms. Indeed, a decreased Parkin expression was reported in lung tissues of COPD patients (Ahmad et al., 2015; Ito et al., 2015). Interestingly, this reduction was correlated with a decrease in lung function in COPD patients (Ito et al., 2015). In our study, we noted the same correlation between protein expression of Parkin and FEV1 in fibroblasts from COPD patient (data not shown). Identification of the mechanism(s) underlying a specific alteration in mitophagy in COPD fibroblasts is beyond the scope of the present study and needs further investigation.

Our results show that, independent from the mechanism underlying impaired mitophagy in COPD, this phenomenon and the associated mitochondrial dysfunction clearly anticipate the pronounced replicative senescence of COPD cells. This is the first demonstration of an association between mitochondrial dysfunction and replicative senescence in COPD. Studies performed in cells exposed to cigarette smoke support a cause–effect relationship between impaired mitophagy, mitochondrial dysfunction, and increased accelerated senescence in COPD. In fact, Ahmad and coworkers (Ahmad et al., 2015) showed that overexpression of Parkin reduces cigarette smoke‐induced DNA damage and cellular senescence when used in combination with a scavenger of mitochondria ROS. In addition, Ito and coworkers demonstrated that the knockdown of *Pink1* or *Parkin* in human bronchial epithelial cells (HBECs) enhanced cellular senescence induced by cigarette smoke exposure (Ito et al., 2015). Our findings showing a protective effect of hemin against both impaired mitophagy and mitochondrial dysfunction and consecutive prevention of replicative senescence further support a cause–effect relationship between the two phenomena in COPD. The role of mitochondrial ROS is also corroborated by the reduction of senescent markers in mitoquinol‐exposed COPD cells.

Hemin could protect against mitochondrial dysfunction by several mechanisms. The first is related to the increased HO‐1 activity and heme‐derived products, since the protective effects of hemin were abolished by two chemically different inhibitors of heme oxygenase activity (ZnPPIX and QC‐15). HO‐1 can induce mitophagy pathways through nuclear respiratory factor‐dependent (NRF‐1‐dependent) expression of the PINK1/PARK2 genes, as demonstrated recently by Suliman, Keenan, and Piantadosi (2017). This effect extends the described activation of mitochondrial biogenesis by CO produced by HO‐1 (Suliman, Carraway, Tatro, & Piantadosi, 2007). Nevertheless, we observed a decreased expression of NRF‐1 in fibroblasts from COPD patients treated with hemin, suggesting that another mechanism could be involved. Indeed, cytoplasmic p53, which accumulates in senescence, interacts with Parkin and prevents its translocation to dysfunctional mitochondria, thus suppressing mitophagy (Ahmad et al., 2015; Hoshino et al., 2013). Similarly, expression of PINK‐1 is low in aging lungs, which participate in the deletion of mitophagy (Bueno et al., 2015). HO‐1 could decrease the accumulation of p53 and release Parkin. Moreover, hemin increased the level of PINK‐1 in COPD fibroblasts, which will participate in the restoration of mitophagy. An additional mechanism could involve the activation of AMPK, which has been shown recently to decrease senescence and inflammation in human bronchial and small airway epithelial cells exposed to cigarette smoke and in a mice model of emphysema (Cheng et al., 2017). Indeed, prophylactic administration of an AMPK activator reduced emphysema, inflammatory responses, and cellular senescence in mice treated with elastase. In our experiments, the expression of AMPK in COPD fibroblasts decreased at senescent stage and was increased by chronic exposures to hemin, suggesting a role of this protein in the attenuation of senescence in COPD fibroblasts (data not shown).

In conclusion, the present results show that HO‐1, through its induction by hemin, protects against mitochondria dysfunction by restoring mitophagy and attenuating senescence in COPD fibroblasts. These results have important implications in terms of a better understanding the mechanisms of cell senescence in COPD. Furthermore, they can set up the basis for the development of new therapeutic strategies in COPD based on counteracting senescence through modulation of HO‐1.

## 4 EXPERIMENTAL PROCEDURES

### 4.1 Materials

Hemin and ZnPPIX were purchased from Sigma (Saint Quentin Fallavier, France) and Frontier Scientific (Distributor: Inochem, Ltd, Carnforth, Lancashire, United Kingdom), respectively. QC‐15 was a kind gift from Prof Kanji Nakatsu from Queen’s University, Kingston, Ontario, Canada. All other reagents were purchased from Sigma unless otherwise stated.

### 4.2 Patients and cells

Primary lung fibroblasts were isolated by the explants technique (Normand & Karasek, 1995) from lung specimens obtained for lung tumor resection from 14 patients with COPD and 13 subjects without clinical, morphologic, or functional signs of COPD (control subjects; Table 1). Classification of COPD severity was based on the 2003 Global Initiative for Chronic Obstructive Lung Disease criteria (Rabe et al., 2007). Informed consent was obtained from all patients, and the study was approved by the “Comité de Protection des Personnes Ile de France IX.”

### 4.3 Cell treatments

In these experiments, fibroblasts were used at a nonsenescent stage (passage three, P3) until a senescent stage (passage seven, P7). We verified in preliminary experiments that the concentration of all pharmacological compounds used in our studies did not alter the viability of cells by using MTT and LDH assays (data not shown).

To evaluate whether chronic exposure of nonsenescent fibroblasts to hemin (an inducer of HO‐1 and substrate for heme oxygenase activity) could prevent replicative senescence in long‐term cultures, fibroblasts were treated with 10 µM hemin every 2 days for 4 weeks. In parallel experiments, the effect of hemin was blocked by daily exposure of cells to 1 µM ZnPPIX, a general inhibitor of heme oxygenase activity, or 15 µM QC‐15, a more selective inhibitor of HO‐1 enzymatic activity. Gene expression levels of HO‐1, protein expression, and heme oxygenase activity were studied. Senescence was characterized by measuring cumulative population doubling levels (PDLs; Holz et al., 2004), senescence‐associated β‐galactosidase activity (SA β‐gal; Dimri, 2005), and the expression of P‐ATM, p21 by immunofluorescence, and p16 by western blot and real‐time quantitative polymerase chain reaction analysis. Markers of inflammation (IL‐6, IL‐8) were assessed by real‐time quantitative polymerase chain reaction analysis.

Analysis of senescence markers, HO‐1 gene, and protein expression and ROS production were performed in all patients, and others analyses were performed in subgroups representative of the whole population.

### 4.4 Measurement of oxidative stress by DCFH‐DA assay and mitochondrial ROS production by mitoSOX red assay

Endogenous ROS were quantified by oxidation of 2′,7′ dichlorofluorescin diacetate (DCFH‐DA) into 2′,7′ dichlorofluorescin (Sigma, Saint Quentin Fallavier, France). Briefly, cells were cultivated in six‐well culture plates and treated either with hemin alone or hemin in the presence of QC‐15 or ZnPPIX. Cells were also treated with 250 µM H_2_O_2_ as a positive control (data not shown). Cells were incubated with 20 µM DCFH‐DA for 30 min at 37°C and fluorescence recorded for 90 min. Results were expressed as a ratio of fluorescence at 90 min over fluorescence at T0.

The detection of mitochondrial superoxide was assessed by oxidation of MitoSOX Red (Life Technologies). Once in the mitochondria, the MitoSOX reagent is oxidized by superoxide and exhibits red fluorescence. The fluorescent product is measured fluorometrically at 580 nm after excitation at 500 nm. Cells were incubated with 5 µM MitoSOX Red for 10 min at 37°C and fluorescence recorded for 15 min. The results were expressed as a ratio of fluorescence at 15 min over fluorescence at T0.

### 4.5 Heme oxygenase activity assay

Cells cultured in 100‐mm diameter petri dishes were collected at passage three or seven after the following treatments: (a) medium alone, (b) hemin (10 μM) added to the culture medium every 2 days, and (c) the combination of hemin and the inhibitors of HO‐1 activity ZnPPIX (1 µM) or QC‐15 (15 µM) added to the medium every day. Samples were incubated with the substrate hemin, NADPH, liver cytosol (a source of biliverdin reductase), and other cofactors to sustain heme oxygenase activity. The reaction was allowed to proceed for 1 hr at 37°C in the dark and was terminated by addition of chloroform to extract the bilirubin produced. Bilirubin was measured spectrophotometrically as described before and calculated in picomoles bilirubin/mg protein/hr (Foresti & Motterlini, 1999).

### 4.6 Cellular bioenergetic analysis using the seahorse bioscience XF analyzer

Analysis of bioenergetic parameters was performed in intact cells using the XF24 analyzer from Seahorse Bioscience (Billerica, MA, USA). Oxygen consumption rate (OCR), which indicates mitochondrial respiration, and extracellular acidification rate (ECAR), an index of glycolysis, were measured in real time in human fibroblasts collected from S‐C or COPD patients after various treatments. Nonsenescent and senescent cells were used for these studies. An optimal cell density of 45,000 cells/well was determined in preliminary experiments. A classical Mito Stress test was performed according to the following procedure: (a) Basal respiration was measured in unbuffered medium; (b) oligomycin (1 µg/ml final concentration), an inhibitor of ATP synthesis, was injected to determine respiration linked to ATP production; (c) the uncoupler carbonyl cyanide 4‐(trifluoromethoxy) phenylhydrazone (FCCP, 0.7 µM final concentration) was added to measure maximal respiration; and (d) rotenone/antimycin A (1 µM final) was applied in combination with block respiration due to simultaneous inhibition of complexes I and III, respectively. Parameters of mitochondrial function were calculated from the respiratory curves as described previously (Wilson et al., 2017).

### 4.7 ATP assay

Cellular ATP levels were assessed at the end of the Seahorse experiments. ATP production was measured using the ATPLite™ bioluminescent assay kit (Perkin Elmer, Courtaboeuf, France) following the manufacturer’s instructions.

### 4.8 Mitochondrial staining

Determination of mitochondrial content was performed using acridine orange 10‐nonyl bromide (NAO), which binds to cardiolipin present in mitochondria. Cells at P3 or P7 subjected to different treatments were fixed for 30 min with 4% paraformaldehyde in PBS at 37°C and incubated for 30 min with 1 µM NAO at 37°C in the dark. After two washes with PBS, cells were analyzed with flow cytometry using a CyAn™ ADP LX7 Analyzer (Beckman Coulter).

### 4.9 Statistical analysis

Data were analyzed with GraphPad Prism 4.0 (La Jolla, CA). Comparisons between groups were performed with Kruskal–Wallis’ nonparametric analysis of variance test followed by two‐by‐two comparisons with Mann–Whitney’s *U* test when a significant difference was detected. *p* < 0.05 was considered statistically significant.

## AUTHOR’S CONTRIBUTION

B.E. involved in cell culture, quantitative PCR studies, biochemical studies, and interpretation of results. S.F.‐K. performed the experiments on mitochondrial function, ATP production and mitochondria content, and interpretation of results. J.‐M.G. performed cell culture, quantitative PCR studies, and interpretation of results. RM carried out design of the study and supervised the project. J.B. carried out design of the study, supervised the project, interpretation of results, and writing of the manuscript. R.F. measured heme oxygenase activity, analyzed data, devised, and supervised the project, and writing of the manuscript. M.D. carried out design of the study, supervised the project, cell culture studies, interpretation of results, and writing of the manuscript.

## References

[acel12837-bib-0001] Ahmad, T. , Sundar, I. K. , Lerner, C. A. , Gerloff, J. , Tormos, A. M. , Yao, H. , & Rahman, I. (2015). Impaired mitophagy leads to cigarette smoke stress‐induced cellular senescence: Implications for chronic obstructive pulmonary disease. FASEB Journal, 29, 2912–2929. 10.1096/fj.14-268276 25792665 PMC4478793

[acel12837-bib-0002] Amsellem, V. , Gary‐Bobo, G. , Marcos, E. , Maitre, B. , Chaar, V. , Validire, P. , … Adnot, S. (2011). Telomere dysfunction causes sustained inflammation in chronic obstructive pulmonary disease. American Journal of Respiratory and Critical Care Medicine, 184, 1358–1366. 10.1164/rccm.201105-0802OC 21885626

[acel12837-bib-0003] Bankier, A. A. , De Maertelaer, V. , Keyzer, C. , & Gevenois, P. A. (1999). Pulmonary emphysema: Subjective visual grading versus objective quantification with macroscopic morphometry and thin‐section CT densitometry. Radiology, 211, 851–858. 10.1148/radiology.211.3.r99jn05851 10352615

[acel12837-bib-0004] Barnes, P. J. (2017). Senescence in COPD and Its Comorbidities. Annual Review of Physiology, 79, 517–539. 10.1146/annurev-physiol-022516-034314 27959617

[acel12837-bib-0005] Bueno, M. , Lai, Y. C. , Romero, Y. , Brands, J. , St Croix, C. M. , Kamga, C. , … Mora, A. L. (2015). PINK1 deficiency impairs mitochondrial homeostasis and promotes lung fibrosis. Journal of Clinical Investigation, 125, 521–538. 10.1172/JCI74942 25562319 PMC4319413

[acel12837-bib-0006] Campisi, J. (2005). Senescent cells, tumor suppression, and organismal aging: Good citizens, bad neighbors. Cell, 120, 513–522. 10.1016/j.cell.2005.02.003 15734683

[acel12837-bib-0007] Carnevali, S. , Petruzzelli, S. , Longoni, B. , Vanacore, R. , Barale, R. , Cipollini, M. , … Giuntini, C. (2003). Cigarette smoke extract induces oxidative stress and apoptosis in human lung fibroblasts. American Journal of Physiology. Lung Cellular and Molecular Physiology, 284, L955–L963. 10.1152/ajplung.00466.2001 12547733

[acel12837-bib-5000] Cheng, X. Y. , Li, Y. Y. , Huang, C. , Li J , Yao, H. W. (2017). AMP-activated protein kinase reduces inflammatory responses and cellular senescence in pulmonary emphysema. Oncotarget. 8(14):22513-22523. 28186975 10.18632/oncotarget.15116 PMC5410241

[acel12837-bib-0008] Church, D. F. , & Pryor, W. A. (1985). Free‐radical chemistry of cigarette smoke and its toxicological implications. Environmental Health Perspectives, 64, 111–126. 3007083 10.1289/ehp.8564111 PMC1568603

[acel12837-bib-0009] Dagouassat, M. , Gagliolo, J. M. , Chrusciel, S. , Bourin, M. C. , Duprez, C. , Caramelle, P. , … Boczkowski, J. (2013). The cyclooxygenase‐2‐prostaglandin E2 pathway maintains senescence of chronic obstructive pulmonary disease fibroblasts. American Journal of Respiratory and Critical Care Medicine, 187, 703–714. 23328527 10.1164/rccm.201208-1361OC

[acel12837-bib-0010] Dimri, G. P. (2005). What has senescence got to do with cancer? Cancer Cell, 7, 505–512. 15950900 10.1016/j.ccr.2005.05.025 PMC1769521

[acel12837-bib-0011] Foresti, R. , & Motterlini, R. (1999). The heme oxygenase pathway and its interaction with nitric oxide in the control of cellular homeostasis. Free Radical Research, 31, 459–475. 10.1080/10715769900301031 10630670

[acel12837-bib-0012] Gispert, S. , Ricciardi, F. , Kurz, A. , Azizov, M. , Hoepken, H. H. , Becker, D. , … Auburger, G. (2009). Parkinson phenotype in aged PINK1‐deficient mice is accompanied by progressive mitochondrial dysfunction in absence of neurodegeneration. PLoS ONE, 4, e5777 10.1371/journal.pone.0005777 19492057 PMC2686165

[acel12837-bib-0013] Hattori, N. , Saiki, S. , & Imai, Y. (2014). Regulation by mitophagy. International Journal of Biochemistry and Cell Biology, 53, 147–150. 10.1016/j.biocel.2014.05.012 24842103

[acel12837-bib-0014] Hoffmann, R. F. , Zarrintan, S. , Brandenburg, S. M. , Kol, A. , de Bruin, H. G. , Jafari, S. , … Heijink, I. H. (2013). Prolonged cigarette smoke exposure alters mitochondrial structure and function in airway epithelial cells. Respiratory Research, 14, 97 10.1186/1465-9921-14-97 24088173 PMC3852998

[acel12837-bib-0015] Holz, O. , Zuhlke, I. , Jaksztat, E. , Muller, K. C. , Welker, L. , Nakashima, M. , … Jorres, R. A. (2004). Lung fibroblasts from patients with emphysema show a reduced proliferation rate in culture. European Respiratory Journal, 24, 575–579. 10.1183/09031936.04.00143703 15459135

[acel12837-bib-0016] Hoshino, A. , Mita, Y. , Okawa, Y. , Ariyoshi, M. , Iwai‐Kanai, E. , Ueyama, T. , … Matoba, S. (2013). Cytosolic p53 inhibits Parkin‐mediated mitophagy and promotes mitochondrial dysfunction in the mouse heart. Nature Communications, 4, 2308 10.1038/ncomms3308 23917356

[acel12837-bib-0017] Hull, T. D. , Boddu, R. , Guo, L. , Tisher, C. C. , Traylor, A. M. , Patel, B. , … George, J. F. (2016). Heme oxygenase‐1 regulates mitochondrial quality control in the heart. JCI Insight., 1, e85817 10.1172/jci.insight.85817 27110594 PMC4838906

[acel12837-bib-0018] Ito, S. , Araya, J. , Kurita, Y. , Kobayashi, K. , Takasaka, N. , Yoshida, M. , … Kuwano, K. (2015). PARK2‐mediated mitophagy is involved in regulation of HBEC senescence in COPD pathogenesis. Autophagy, 11, 547–559. 10.1080/15548627.2015.1017190 25714760 PMC4502689

[acel12837-bib-0019] Lerner, C. A. , Sundar, I. K. , & Rahman, I. (2016). Mitochondrial redox system, dynamics, and dysfunction in lung inflammaging and COPD. International Journal of Biochemistry and Cell Biology, 81, 294–306. 10.1016/j.biocel.2016.07.026 27474491 PMC5154857

[acel12837-bib-0020] Maestrelli, P. , El Messlemani, A. H. , De Fina, O. , Nowicki, Y. , Saetta, M. , Mapp, C. , & Fabbri, L. M. (2001). Increased expression of heme oxygenase (HO)‐1 in alveolar spaces and HO‐2 in alveolar walls of smokers. American Journal of Respiratory and Critical Care Medicine, 164, 1508–1513. 10.1164/ajrccm.164.8.2011083 11704604

[acel12837-bib-0021] Mannino, D. M. , & Buist, A. S. (2007). Global burden of COPD: Risk factors, prevalence, and future trends. Lancet, 370, 765–773. 17765526 10.1016/S0140-6736(07)61380-4

[acel12837-bib-0022] Matsuda, N. , Sato, S. , Shiba, K. , Okatsu, K. , Saisho, K. , Gautier, C. A. , … Tanaka, K. (2010). PINK1 stabilized by mitochondrial depolarization recruits Parkin to damaged mitochondria and activates latent Parkin for mitophagy. Journal of Cell Biology, 189, 211–221. 10.1083/jcb.200910140 20404107 PMC2856912

[acel12837-bib-0023] Normand, J. , & Karasek, M. A. (1995). A method for the isolation and serial propagation of keratinocytes, endothelial cells, and fibroblasts from a single punch biopsy of human skin. In Vitro Cellular and Developmental Biology – Animal, 31, 447–455. 10.1007/BF02634257 8589888

[acel12837-bib-0024] Noureddine, H. , Gary‐Bobo, G. , Alifano, M. , Marcos, E. , Saker, M. , Vienney, N. , … Adnot, S. (2011). Pulmonary artery smooth muscle cell senescence is a pathogenic mechanism for pulmonary hypertension in chronic lung disease. Circulation Research, 109, 543–553. 10.1161/CIRCRESAHA.111.241299 21719760 PMC3375237

[acel12837-bib-0025] Nyunoya, T. , Monick, M. M. , Klingelhutz, A. L. , Glaser, H. , Cagley, J. R. , Brown, C. O. , … Hunninghake, G. W. (2009). Cigarette smoke induces cellular senescence via Werner's syndrome protein down‐regulation. American Journal of Respiratory and Critical Care Medicine, 179, 279–287. 10.1164/rccm.200802-320OC 19011155 PMC2643077

[acel12837-bib-0026] Palacino, J. J. , Sagi, D. , Goldberg, M. S. , Krauss, S. , Motz, C. , Wacker, M. , … Shen, J. (2004). Mitochondrial dysfunction and oxidative damage in parkin‐deficient mice. Journal of Biological Chemistry, 279, 18614–18622. 14985362 10.1074/jbc.M401135200

[acel12837-bib-0027] Piantadosi, C. A. , Withers, C. M. , Bartz, R. R. , MacGarvey, N. C. , Fu, P. , Sweeney, T. E. , … Suliman, H. B. (2011). Heme oxygenase‐1 couples activation of mitochondrial biogenesis to anti‐inflammatory cytokine expression. Journal of Biological Chemistry, 286, 16374–16385. 10.1074/jbc.M110.207738 21454555 PMC3091243

[acel12837-bib-0028] Rabe, K. F. , Hurd, S. , Anzueto, A. , Barnes, P. J. , Buist, S. A. , Calverley, P. , … Zielinski, J. (2007). Global strategy for the diagnosis, management, and prevention of chronic obstructive pulmonary disease: GOLD executive summary. American Journal of Respiratory and Critical Care Medicine, 176, 532–555. 10.1164/rccm.200703-456SO 17507545

[acel12837-bib-0029] Suliman, H. B. , Carraway, M. S. , Tatro, L. G. , & Piantadosi, C. A. (2007). A new activating role for CO in cardiac mitochondrial biogenesis. Journal of Cell Science, 120, 299–308. 10.1242/jcs.03318 17179207

[acel12837-bib-0030] Suliman, H. B. , Keenan, J. E. , & Piantadosi, C. A. (2017). Mitochondrial quality‐control dysregulation in conditional HO‐1‐/‐ mice. JCI Insight, 2, e89676 10.1172/jci.insight.89676 28194437 PMC5291731

[acel12837-bib-0031] Tsuji, T. , Aoshiba, K. , & Nagai, A. (2010). Alveolar cell senescence exacerbates pulmonary inflammation in patients with chronic obstructive pulmonary disease. Respiration, 80, 59–70. 10.1159/000268287 20016134

[acel12837-bib-0032] Wilson, J. L. , Bouillaud, F. , Almeida, A. S. , Vieira, H. L. , Ouidja, M. O. , Dubois‐Rande, J. L. , … Motterlini, R. (2017). Carbon monoxide reverses the metabolic adaptation of microglia cells to an inflammatory stimulus. Free Radical Biology and Medicine, 104, 311–323. 10.1016/j.freeradbiomed.2017.01.022 28108277

[acel12837-bib-0033] Yoshida, T. , & Tuder, R. M. (2007). Pathobiology of cigarette smoke‐induced chronic obstructive pulmonary disease. Physiological Reviews, 87, 1047–1082. 10.1152/physrev.00048.2006 17615396

