# Supplementary material for: Heme oxygenase‐1 induction attenuates senescence in chronic obstructive pulmonary disease lung fibroblasts by protecting against mitochondria dysfunction
Source: Aging Cell. 2018 Oct 19;17(6):e12837. doi: 10.1111/acel.12837 (PMC6260925; doi:10.1111/acel.12837)
Supplement: Supplementary file 1 [file ACEL-17-e12837-s001.pdf]

**Supplementary-1 (S1)**

**A**

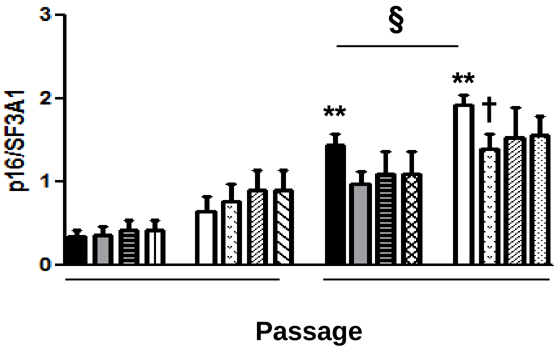


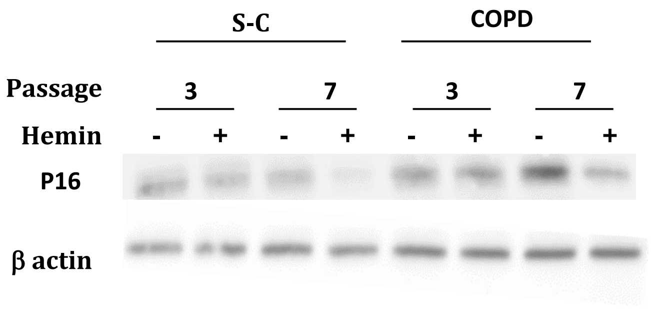

**Supplementary-2 (S2)**

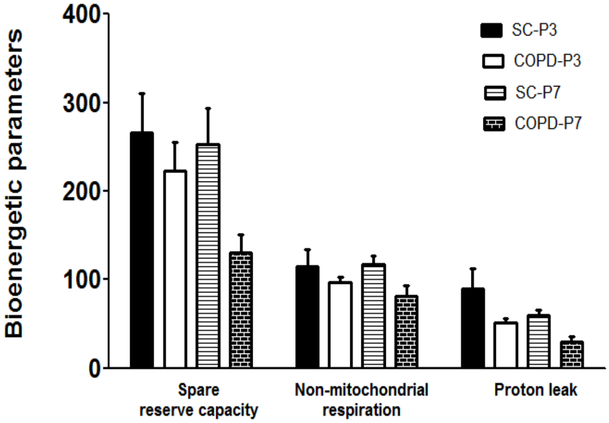

**Supplementary-3 (S3)**

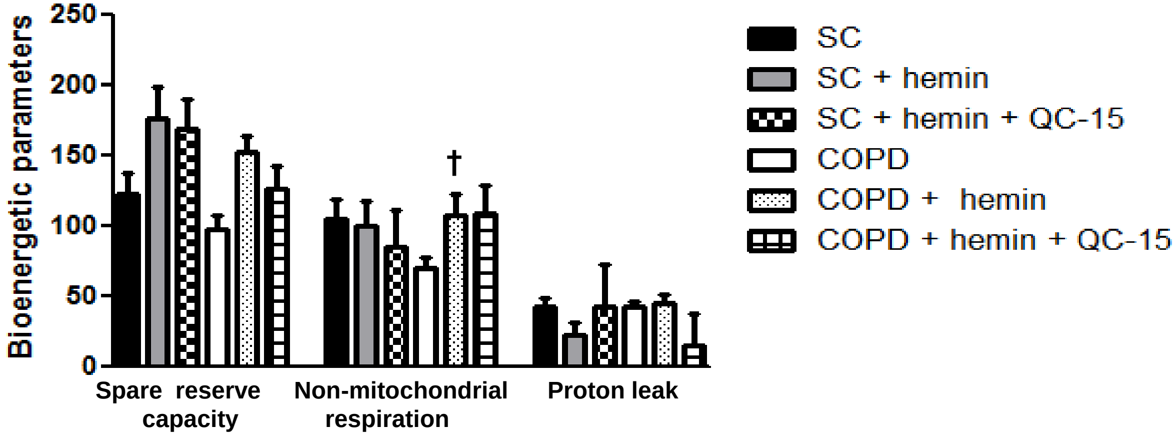

Supplementary-4 (S4)

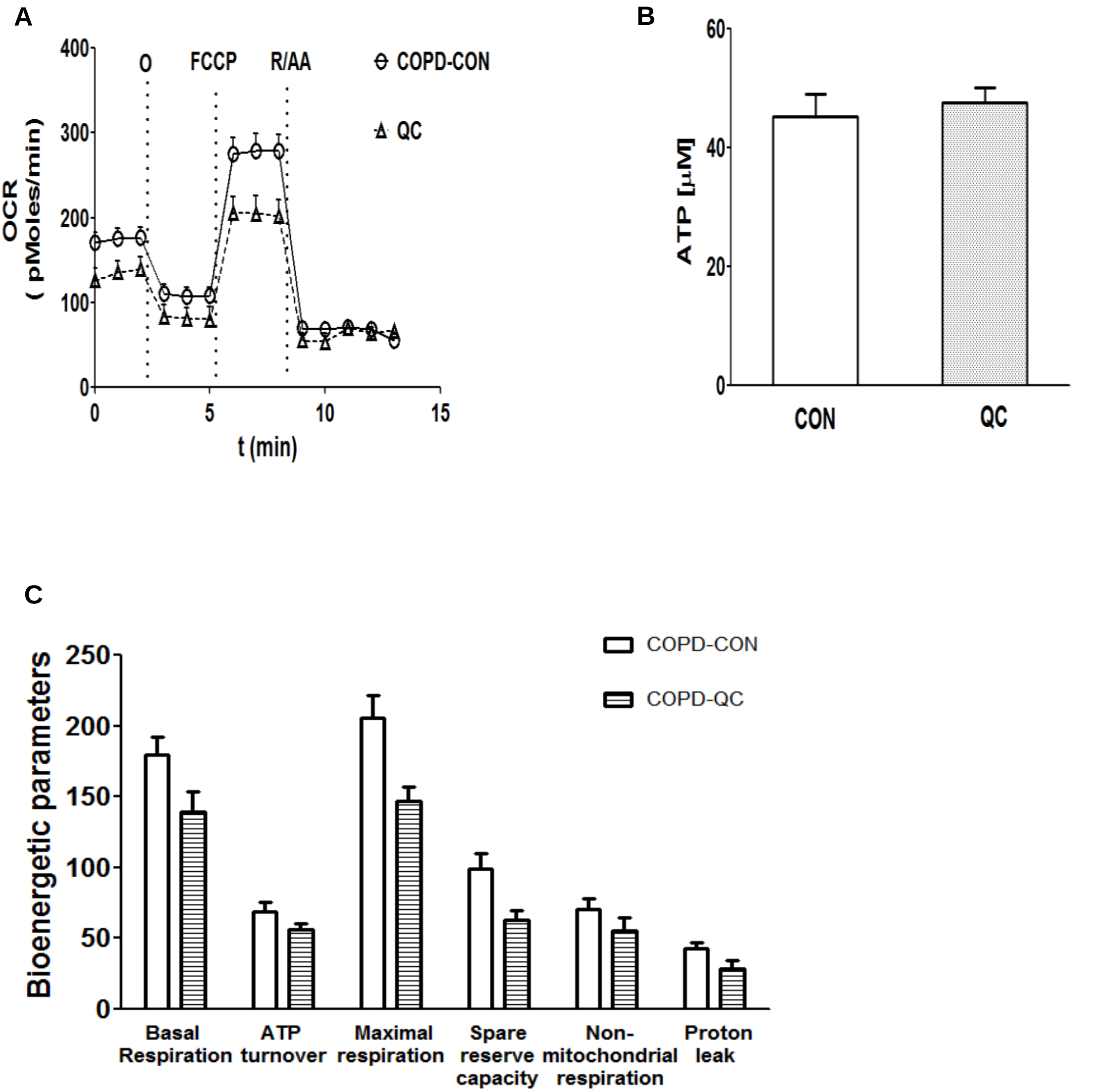

## Supplementary-5 (S5)

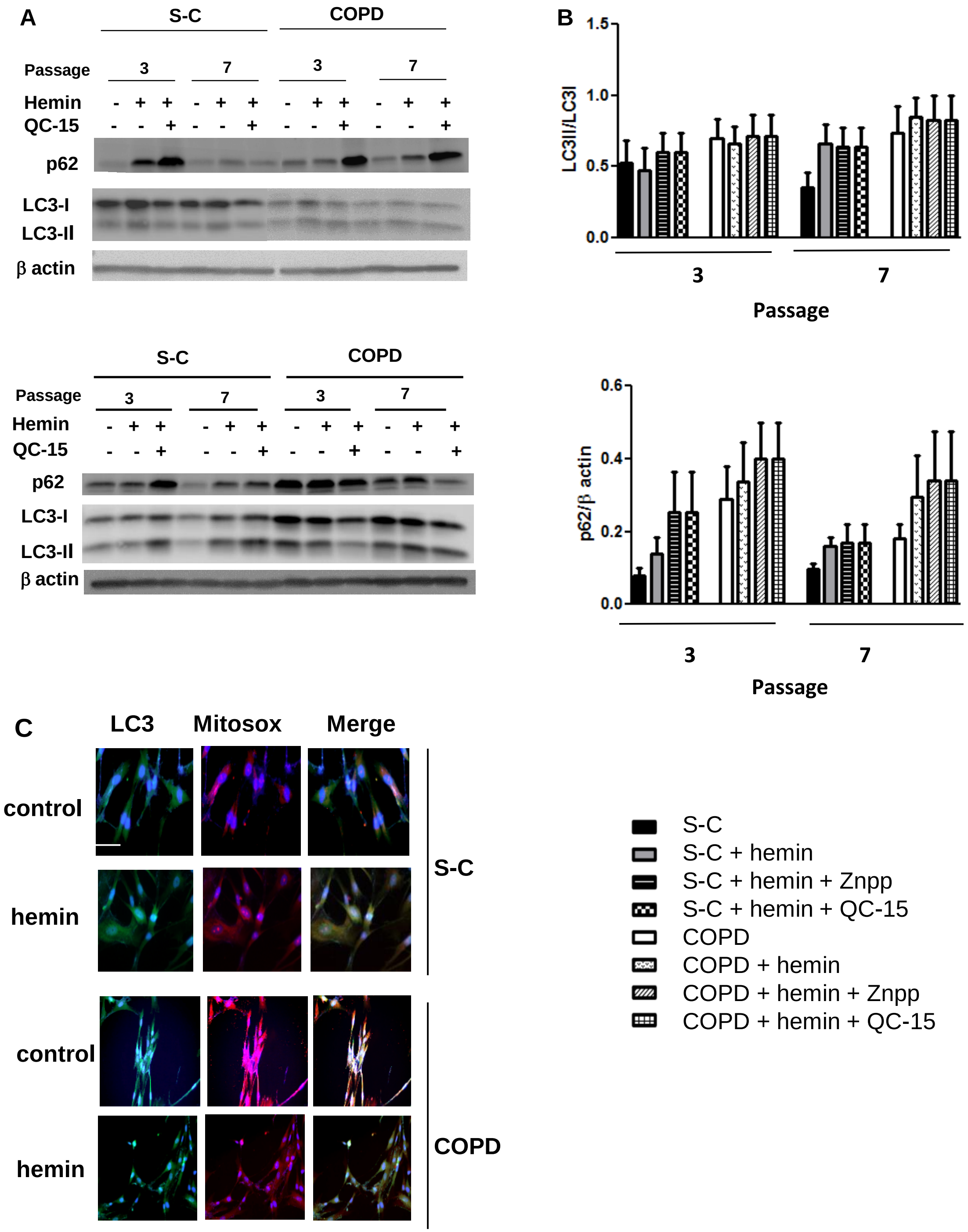

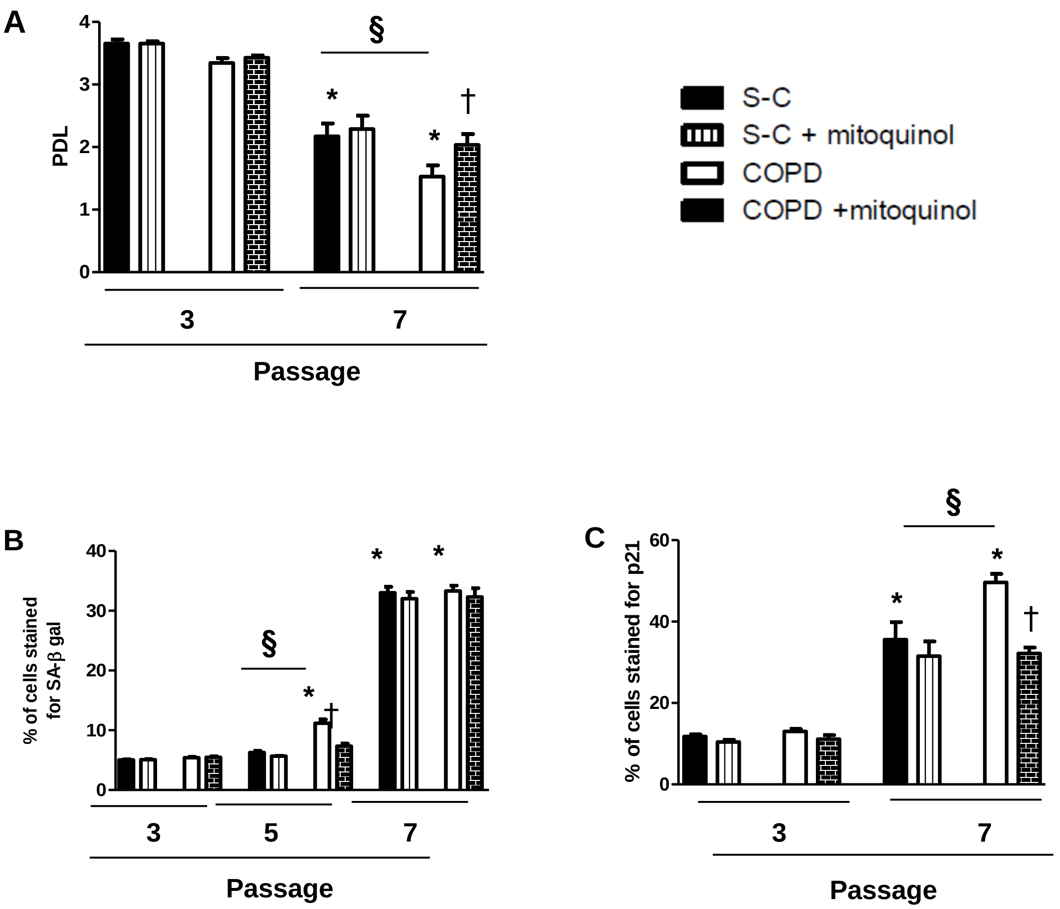
A
4
3
2
1
0
PDL
§
*
†
*
3
7
Passage
S-C
S-C + mitoquinol
COPD
COPD +mitoquinol
B
40
30
20
10
0
% of cells stained for SA-β gal
§
*
†
*
*
3
5
7
Passage
C
60
40
20
0
% of cells stained for p21
§
*
*
†
3
7
Passage
