# Supplementary material for: Heme oxygenase‐1 induction attenuates senescence in chronic obstructive pulmonary disease lung fibroblasts by protecting against mitochondria dysfunction
Source: Aging Cell. 2018 Oct 19;17(6):e12837. doi: 10.1111/acel.12837 (PMC6260925; doi:10.1111/acel.12837)
Supplement: Supplementary file 2 [file ACEL-17-e12837-s002.doc]

**Online Data Supplement**

**HO-1 INDUCTION PREVENTS SENESCENCE IN COPD LUNG FIBROBLASTS BY PROTECTING AGAINST MITOCHONDRIA DYSFUNCTION**

Benjamin Even ^2^, Sarah Fayad-Kobeissi ^3^, Jean- Marie Gagliolo ^2^, Roberto Motterlini ^3^, Jorge Boczkowski ^1,2,4^, Roberta Foresti ^3*^, Maylis Dagouassat ^1,2*^

**Patients**

Primary lung fibroblasts were isolated by the explant technique (1) from specimens obtained for lung tumor resection from 14 patients with COPD and 13 subjects without clinical, morphological or functional signs of COPD (smoker controls, S-C) (Table 1). Classification of COPD severity was based on the 2003 Global initiative for chronic obstructive lung disease (GOLD) criteria (ref). Informed consent was obtained from all patients and the study was approved by the “Comité de Protection des Personnes Ile de France IX”.. None of the patients were treated with corticosteroids, bronchodilatator therapy, or had a history of cancer chemotherapy or radiotherapy.

**Isolation of pulmonary fibroblasts and culture**

Lung fibroblasts were isolated from patients undergoing resective surgery for pulmonary carcinoma. Pleura-free parenchymal specimens were excised after careful macroscopic evaluation from peripheral areas of the lobe as far away as possible from the tumor site. Lung fibroblasts were obtained by the explant method (Normand and Karasek, 1995). Cells were cultured in Dulbecco’s modified Eagle’s medium (DMEM) (Invitrogen, Cergy-Pontoise, France) containing 10% heat-inactivated fetal bovine serum (FBS) (PAA, Les Mureaux, France), 100 U/ml penicillin, 100 µg/ml streptomycin (Invitrogen, Cergy-Pontoise, France), at 37°C in a 5% CO_2_ atmosphere. Isolated cells were characterized as fibroblasts by morphological appearance and by immunocytochemical assessment of the expression pattern of specific proteins. Cells stained positive with anti-vimentin and always stained negative with anti-pancytokeratin and anti-CD31 (Dako Cytomation, Trappes, France). Fibroblasts were negative for the cancer associated fibroblasts markers PDGF-Rβ (Abcam, Paris, France), desmin (Dako Cytomation, Trappes, France) and fibroblast activation protein α (FAP-α) (Clinisciences, Montrouge, France) (data not shown). All cultures contained between 5 to 7% of α smooth muscle actin positive cells (Invitrogen, Cergy-Pontoise, France).

**Gene expression analysis of HO-1, genes involved in mitochondrial biogenesis, senescence markers and inflammatory mediators**

Total RNA was extracted with an RNeasy kit (Qiagen, Courtaboeuf, France) according to manufacturer’s instructions. The gene expression level of HO-1, the expression of the mitochondrial biogenesis genes nuclear respiratory factor 1 (NRF1) and peroxisome proliferator-activated receptor gamma coactivator -1α (PGC-1α), the expression of the senescence markers p16 and p21 and the inflammatory cytokines IL-6, IL-8 was analyzed by RT-qPCR using a lightcycler and expressed as the ratio to a house-keeping gene (S3FA1). In fibroblasts from COPD and smoker patients, the gene expression of SF3A1 is constant whatever the fibroblasts or the treatments considered. Primer sequences are given in Table

**Staining for Senescence-associated β-Galactosidase (SA β-Gal)**

Pulmonary fibroblasts were cultivated in an 8-well glass slide chamber. After exposure to different compounds, staining for SA β-Gal activity (Ozyme, Saint-Quentin-en-Yvelines, France) was performed as described (Dimri, 2005). Cells were fixed in 2% formaldehyde and 0.2% glutaraldehyde for 10 minutes at room temperature. The slides were then rinsed with PBS and incubated with an SA β-gal staining solution containing 40 mM sodium citrate (pH 6), 150 mM, NaCl, 5 mM potassium ferricyanide, 5 mM potassium ferrocyanide, 2 mM MgCl_2_ and 1 mg/ml 5 bromo-4-chloro-3-indolyl-β-D-galactoside. Positive cells stained with blue were counted under visible light by two independent observers.

**Preparation of cell homogenates for Western blot analysis**

Lung fibroblasts samples were lysed on ice in a lysis buffer (10 mM Tris-HCl, pH 6.8, 150 mM NaCl, 10 mM Hepes, Saccharose 500 mM, Na_2_ EDTA 1 mM, 1.0% NP-40, 10% anti-protease and 1% anti-phosphatase). The protein concentration of these cell extracts was quantified using Bradford protein assay (Bio-Rad Laboratories, Marne-La-Coquette, France). An equal amount of protein (40 µg/lane) from each cell extract was resolved on a 10% or 12% SDS-PAGE gel. Proteins were blotted to an Immuno-Blot polyvinylidenediflouride (PVDF) membrane (Bio-Rad Laboratories, Marne-La-Coquette, France) by electrophoresis. The membranes were blocked with TBS-T blocking buffer (10 % milk in 25 mM, Tris–HCl, pH 7.4, 3 mM; KCl, 140 mM; NaCl, and 0.05% Tween) and subsequently probed with the following primary antibodies either overnight at 4°C: mouse monoclonal PINK1 (1:1000, Abcam, Paris, France), rabbit polyclonal Parkin (1:1000 Abcam, Paris, France); rabbit polyclonal LC3I and II (1:1000, Sigma, Saint Quentin Fallavier, France), and polyclonal guinea pig p62 (1:1000, Progen, Paris, France), monoclonal mouse SIRT3 (1:1000, Santa Cruz, Clinisciences, Montrouge, France), monoclonal rabbit AMPK (1:1000, Cell Signaling, Saint-Quentin-en-Yvelines, France), polyclonal rabbit p-AMPK (1:1000, Cell Signaling, Saint-Quentin-en-Yvelines, France) and 1 h at room temperature for rabbit polyclonal HO-1 (1:1000, Enzo Life Science, Villeurbanne, France). After extensive washing with TBS-T, immunoblots were then incubated with an appropriate peroxidase-conjugated secondary antibody (GE Healthcare Europe, Orsay, France) for 1 h at room temperature. After three washes with TBS-T, immunoblots were detected using the ECL Western Blotting Detection Reagents (GE Healthcare Europe, Orsay, France) and recorded by exposure of the immunoblots to an X-ray film (Sigma, Saint Quentin Fallavier, France). The results were expressed as a ratio to β-actin expression. With this aim, immunoblots were incubated with β-actin primary antibody for 30 minutes at room temperature (Sigma, Saint Quentin Fallavier, France). After washes, immunoblots were incubated with alkaline phosphatase-conjugated secondary antibody (Bio-Rad Laboratories, Marne-La-Coquette, France) for 30 minutes at room temperature. Finally, immunoblots were revealed using the Immun-Star AP Substrate (Bio-Rad Laboratories, Marne-La-Coquette, France). X-ray films were quantified by using Image J software (NIH, USA)

**Cell treatments**

We treated our cells with mitoquinol (an antioxidant that accumulates in mitochondria). We checked in preliminary experiments that the concentration of this pharmacological compound used in our studies did not alter the viability of cells by using MTT and LDH assays (data not shown). To evaluate whether chronic exposure of non-senescent fibroblasts to mitoquinol could prevent replicative senescence in long-term cultures, fibroblasts were treated with 20 nM of mitoquinol every two days for 4 weeks. Senescence was characterized by measuring population doubling levels (PDLs), senescence-associated β-galactosidase activity (SA β-gal), and the expression of p21 by immunofluorescence.

**Co-immunostaining**

In order to evaluate the mitophagy, we used a co-staining with an autophagosome marker (LC3) and a mitochondrial marker (MitoSOX Red (mitochondrial superoxide marker). Fibroblasts were cultivated in 8-well glass slide chamber. After treatments with hemin, cells stained were incubated with 5µM of MitoSOX Red for 10 minutes at 37°C. Cells were fixed in 4% paraformaldehyde for 15 min at 4°C and blocked in 2% BSA at ambient temperature. Cells were incubated with following primary antibodie overnight at 4°C: rabbit polyclonal LC3 (1:200, Sigma, Saint Quentin Fallavier, France), Fluorescence signal was detected by using a goat anti- rabbit secondary antibody (1:500, conjugated with Alexa 594, Invitrogen, Cergy-Pontoise, France). The slides were mounted by using prolong DAPI (Invitrogen, Cergy-Pontoise, France). A fluorescence microscope coupled to a digital camera utilizing axiovision software was used to view and acquire images (Zeiss, Jena, Germany).

**References**

Dimri, G. P. (2005). What has senescence got to do with cancer? *Cancer Cell* **7,** 505-512.

Normand, J., and Karasek, M. A. (1995). A method for the isolation and serial propagation of keratinocytes, endothelial cells, and fibroblasts from a single punch biopsy of human skin*. In Vitro Cell Dev Biol Anim* **31**, 447-455.

**Table**: **Real-time PCR primer sequences**

| **Human genes** | **Forward** | **Reverse** |
| --- | --- | --- |
| **p16** | GGGTCGGGTAGAGGAGGTG | CATCATGACCTGGATCGGC |
| **p21** | GAGACTCTCAGGGTCGAAAACG | GGATTAGGGCTTCCTCTTGGA |
| **IL6** | AGCCACTCACCTCTTCAGAACGAA | AGTGCCTCTTTGCTGCTTTCACAC |
| **IL8** | CAGAGACAGCAGAGCACACA | GATGGTTCCTTCCGGTGGTT |
| **NRF1** | CTGCAGGAAACTTCGAGCCA | GTTCTGCCAGAGCAGACTCC |
| **PGC1α** | TTGAAAAAGCTTGACTGGCGT | GTCTTCACCAACCAGAGCAG |
| **HO-1** | GCTGACCCATGACACCAAGG | AGTGTAAGGACCATCGGAGA |
| **SF3A1** | TGCAGGATAAGACGGAATGGAAACTGA | GTAGTAAGCCAGTGAGTTGGAATCTTTG |

**Figures legends**

**Figure S1: Gene and protein expression of senescent markers.** Fibroblasts from COPD patients (n=16) and smoker controls (S-C, n=13) were treated chronically with either hemin (10 µM) alone or with the inhibitors of HO-1 activity QC-15 (15 µM) for 4 weeks. Transcriptional expression of p16 **(A)** by real time qPCR in pulmonary fibroblasts. Protein expression of p16 obtained by western blot. **(B**) Protein expression of p21 obtained by western blot. Data are presented as mean ± SEM. ** p<0.01 passage 7 (senescent stage) vs. passage 3 (non-senescent stage), **†** *p*<0.05 cells treated with hemin vs. cells treated with DMSO, **§** p<0.05 COPD vs. S-C.

**Figure S2: Bioenergetics Parameters in fibroblasts at non-senescent and senescent stage:** Fibroblasts from COPD patients (n=16) and smoker controls (S-C, n=13) were cultured for 4 weeks to reach senescent state. Bioenergetics parameters (spare reserve capacity, non-mitochondrial respiration and proton leak are presented in this Figure. These bioenergetics parameters were calculated from the results obtained with the Mito Stress assay performed using the Seahorse XF analyzer in fibroblasts at non senescent stage (passage 3 (P3)) and senescent stage (passage 7 (P7)).

**Figure S3: Bioenergetics Parameters in fibroblasts at senescent stage treated chronically with hemin.** Fibroblasts from COPD patients (n=16) and smoker controls (S-C, n=13) were treated chronically with either hemin (10 µM) alone or with the inhibitor of HO-1 activity QC-15 (15 µM) for 4 weeks. Bioenergetics parameters (spare reserve capacity, non-mitochondrial respiration and proton leak are presented in this Figure. These bioenergetics parameters were calculated from the results of the Mito Stress assay performed using the Seahorse XF analyzer. **†** *p*<0.05 cells treated with hemin vs. cells treated with DMSO.

**Figure S4**: **Bioenergetics Parameters in fibroblasts from COPD patients at senescent stage treated chronically with QC-15.** Fibroblasts from COPD patients (n=6) were treated chronically with QC-15 (15 µM) for 4 weeks. Bioenergetics parameters (A, C) calculated from the results of the Mito Stress assay performed using the Seahorse XF analyzer. B) ATP levels.

# Figure S5: Hemin did not modify the autophagic flow, but restored mitophagy. Fibroblasts from COPD patients and smoker controls (S-C) were treated chronically with either hemin (10 µM) alone or in the presence of inhibitors of HO-1 activity: ZnPPIX (1 µM) or QC-15 (15 µM) for 4 weeks. A, B) Analysis and quantification of LC3II, I and p62 protein levels by western blot. C) Immunocytochemical expression of LC3 (green fluorescence). Mitochondria were identified by MitoSOX Red. Nuclei were counterstained with DAPI. Scale bar = 20 µM. Data are presented as mean ± SEM.

**Figure S6: Mitoquinol attenuated replicative senescence in COPD fibroblasts. (A)** Fibroblasts from COPD patients (n = 5) and smoker controls (S-C, n= 5) were treated chronically with mitoquinol (20 nM) for 4 weeks. **A)** The rate of proliferation was evaluated by the population doubling level (PDL). **B)** Percentage of Senescence associated (SA) β-Gal-positive cells. **C)** Quantification of p21 obtained by immunostaining. Data are presented as mean ± SEM in the whole Figure. ***** *p*<0.05, passage 7 (senescent stage) vs. passage 3 (non senescent stage), **†** *p*<0.05 cells treated with mitoquinol vs. cells treated with DMSO, **§** p<0.05 COPD vs. S-C.
